# Editorial: Decoding the genome of acute lymphoblastic leukemia through genomic and transcriptomic approaches

**DOI:** 10.3389/fonc.2024.1368676

**Published:** 2024-02-06

**Authors:** Silvia Jiménez-Morales, Augusto Rojas-Martinez, Gisela Barbany

**Affiliations:** ^1^ Laboratorio de Innovación y Medicina de Precisión, Núcleo “A”, Instituto Nacional de Medicina Genómica, Mexico City, Mexico; ^2^ Tecnologico de Monterrey, The Institute for Obesity Research and Escuela de Medicina y Ciencias de la Salud, Monterrey, Nuevo León, Mexico; ^3^ Department of Clinical Genetics, Karolinska University Hospital, Stockholm, Sweden; ^4^ Department of Molecular Medicine and Surgery and Center for Molecular Medicine, Karolinska Institutet, Stockholm, Sweden

**Keywords:** acute lymphoblastic leukemia, RNA-Seq - RNA sequencing, iAMP21, whole exome sequencing, fusion genes, fish

Leukemia is a group of life-threatening malignant disorders of the blood and bone marrow that strikes subjects of all ages and has disparities in its incidence and mortality rates among countries and ethnicities (1, 2). Overall, among all types of leukemia, acute lymphoblastic leukemia (ALL) is the leading cause of hematological malignancies in children (2, 3), with more aggressive disease biology and worse tolerability of treatment strategies and outcomes in adults than their younger counterparts (4). ALL is characterized by a highly complex abnormal genomic background (i.e., aneuploidies, point mutations, and copy number alterations), but the most relevant is the presence of fused genes with clinical significance (3, 5). The genome mapping project, alongside the rapid growth of high-throughput technologies, has increased our knowledge of the genomic background of ALL, giving us the opportunity to identify novel genetic lesions. Several of these lesions have been reported to impact the likelihood of treatment response, disease-free survival (DFS) (5), and ultimately the overall survival (OS) of patients with this malignancy. Nevertheless, short- or long-term side effects of chemotherapy and the rising cost involved in cancer treatment remain important challenges. There is mounting evidence that decoding the genome of childhood and adulthood ALL might help to identify new therapeutic targets and prognostic markers to develop optimal treatment strategies for these patients, reduce the toxicity of conventional chemotherapy, and improve outcomes. Based on the scope of this Research Topic, four original research articles explored the genome and transcriptome to identify new biomarkers in ALL or better tools to detect them.

Fang et al. found a novel immunoglobulin heavy chain (IGH) rearrangement in B-ALL patients with the P2Y purinoceptor 8 (P2RY8) locus by integrating fluorescence *in situ* hybridization (FISH), transcriptome sequencing, and RT–qPCR techniques. RNA sequencing detected a rearrangement involving the *P2RY8* and *IGH* loci. In the three patients, the breakpoint in *P2RY8* was located in intron 1, resulting in the fusion of exon 1 to different segments of the IGH locus, which all resulted in the overexpression of a truncated *P2RY8*, not present in healthy donor samples or other B-ALL samples. The authors suggest that the pathogenicity of *P2RY8/IGH* rearrangement is mediated by the overexpression of the truncated *P2RY8* transcripts. Thus, *P2RY8* gene extends the more than 40 genes involved in fusions, and *P2RY8/IGH* fusion gene emerges as a potential molecular marker to evaluate measurable residual disease.

The intrachromosomal amplification of chromosome 21 (iAMP21) defines a rare high-risk subtype of ALL that accounts for approximately 2% of all B-cell precursor ALL (BCP-ALL) cases. This rearrangement is commonly detected by copy number analysis using single-nucleotide polymorphism (SNP) arrays or by metaphase FISH using the probe designed to detect the *ETV6*–*RUNX1* gene fusion. The amplified region contains coding and non-coding genes that might contribute to the leukemogenesis process, but the underlying driver lesion in iAMP21 ALL has not been completely elucidated. In their study, Horman et al. combined comparative genomic hybridization and gene expression microarrays in a series of 64 iAMP21 ALL patients to narrow the size of the common amplified region (CAR) and to identify the critical genes located in this segment, and they used additional reported cases to verify their findings. The authors found a minimal CAR of 1.57 Mb in all iAMP21-positive cases, which involved 12 protein-coding genes (PGCs) and one non-coding gene. Interestingly, the *RUNX1* locus currently used to define iAMP21 was not included in the CAR. The study also identified *RIPPLY3*, a transcriptional co-repressor not previously associated with leukemogenesis, as the gene whose transcriptional signature showed the largest overlap with the iAMP21 transcriptional signature among the CAR overexpressed genes. Based on their finding, the authors propose a relevant role of *RIPPLY3* in the pathogenesis of iAMP21 and propose a new definition of iAMP21 ALL to include those with low *RUNX1* amplification.

As we know, the identification of biomarkers is critical to elucidate outcomes associated with and to develop appropriate treatment strategies in ALL patients. In their report, Wu et al. described two cases of pediatric ALL harboring a TCF4::ZNF384 fusion identified through the RNA-Seq technique among a series of pediatric ALL with normal karyotypes. Notably, *TCF4* gene represents a new partner of *ZNF384*. The incidence of *ZNF384* rearrangements of ALL in children is low; however, ZNF384 rearrangement ALL has been proposed to represent a distinct subtype of ALL. As these rearrangements are difficult to detect with karyotype analysis and are not routinely sought by other methods, the true incidence has not yet been established. Thus, this paper highlights the relevance of using throughput sequencing tools to detect cryptic fusion genes.

Rezayee et al., in an interesting study, demonstrated that whole-genome sequencing (WGS) is suitable to replace current multimodal methods to detect clinically relevant aberrations that are mandatory for current treatment protocol in children with B-cell ALL. To achieve this goal, classified and unclassified bone marrow samples of ALL patients were included. Additionally, the authors investigated whether sequencing depth could affect variant detection. Samples were down-sampled *in silico* from 90X to 30X coverage. This important study showed that WGS with a sequencing depth of 30X is a powerful method to identify the known clinically relevant abnormalities such as point mutations, indels, copy number alterations, and aneuploidies and enables the detection of emerging lesions that define new genetic subtypes of ALL.

In conclusion, ALL represents a group of clinically heterogeneous hematologic malignancies with high genomic complexity and outcome diversity. The stratification of the patients according to genetic risk among others has contributed to improved outcomes, and thus, genetic characterization of ALL is part of the investigations performed at diagnosis. Detecting new biomarkers as potentially targetable is becoming highly relevant in this field. Thus, this Research Topic highlights advances in the understanding of critical aspects of the biology of leukemia to facilitate the development of effective diagnostic and therapeutic strategies for improved patient outcomes. As Guest Editors for this Research Topic, we hope that the readers enjoy reading these outstanding papers.

## Author contributions

SJ-M: Conceptualization, Investigation, Writing – original draft, Writing – review & editing. AR-M: Writing – review & editing. GB: Writing – review & editing.
